# Controlled Synthesis of SnO_2_ Nanocrystals with Tunable Band Gaps

**DOI:** 10.1021/prechem.4c00107

**Published:** 2025-03-17

**Authors:** Can Li, Xin Shu, Jun Zhang, Joseph Delgado, Prabhu Bharathan, Yuxuan Wang, Chenyu Wang, Jiye Fang

**Affiliations:** † Department of Chemistry, 14787 State University of New York at Binghamton, Binghamton, New York 13902, United States; ‡ School of Materials Science and Engineering, 71136 China University of Petroleum (East China), Qingdao 266580, China; § Materials Science and Engineering Program, State University of New York at Binghamton, Binghamton, New York 13902, United States

**Keywords:** tin oxide, band gap, nanocrystal, oxygen defect, optical absorption

## Abstract

Tin­(IV) oxide nanocrystals (SnO_2_ NCs) have significant potential in various applications, with their performance closely related to their band gap. The band gap is influenced by the size and shape of the NCs, which can be precisely controlled by adjusting reaction conditions. In this study, we present deliberately designed synthesis protocols to produce high-quality SnO_2_ NCs with tunable band gaps using different methods. Key factors affecting the synthesis include control of the oxidizing agent, reaction temperature, solvent selection, and reaction time optimization. The resulting NCs were characterized by using TEM, XRD, XPS, and optical spectroscopy. Notably, SnO_2_ NCs synthesized by controlling the oxidizing agent (air injection) in a hot organic solution were smaller in size and exhibited abundant oxygen vacancies. In contrast, extending the reaction time or using ethanol as a solvent in hydrothermal systems facilitated larger spherical or rod-like SnO_2_ NCs with fewer oxygen vacancies. Further analysis of the band gap and valence band maximum energy revealed that the abundant vacancies in SnO_2_ NCs synthesized with the air-controlled hot organic solution method resulted in a narrower band gap and an upshifted valence band. These synthetic strategies illustrate the potential for deliberately designing SnO_2_ NCs with optimized electronic structures for various applications.

## Graphical abstract

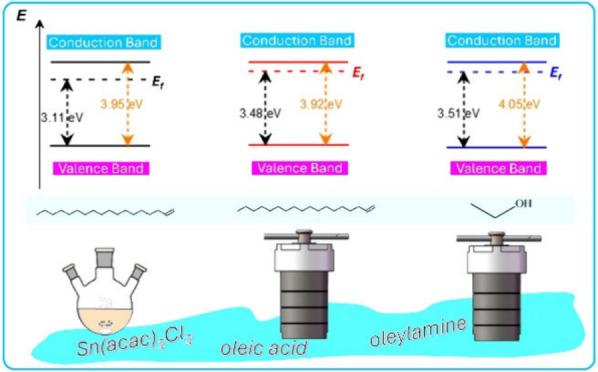

## Introduction

Due to their unique electronic structures and excellent characteristics at the nanoscale, metal oxide nanocrystals (NCs), such as titanium dioxide (TiO_2_), zinc oxide (ZnO), tin­(IV) oxide (SnO_2_), and cerium­(IV) oxide (CeO_2_), have found widespread applications in various fields, including biomedicine and healthcare,^1–3^ energy storage and conversion (*e.g.*, lithium batteries and fuel cells),^4–7^ light emitting diode (LED) devices,^8–12^ and catalysis.^13–17^ Among these, SnO_2_, with its tetragonal rutile structure (*P*4_2_/*mnm*), is well-known as an n-type semiconductor with a wide band gap of 3.6 eV (at 300 K) and has attracted significant interest, owing to its diverse potential applications.^18–27^ For example, because SnO_2_ exhibits well-aligned conduction band minima with halide perovskites, along with outstanding electron mobility and excellent stability against chemical degradation at the interface, SnO_2_ has been recognized as an ideal candidate for charge-transport layers in high-performance perovskite solar cells.^28–31^ To further enhance both the performance and processability of SnO_2_ as an electron transport layer, it is crucial to develop nanoscaled SnO_2_ with controlled internal and external defects of photoelectrodes.

Several wet-chemistry approaches have been employed to synthesize SnO_2_ NCs, including hydrothermal processes, nonaqueous hot-solution methods, and microwave-irradiation techniques.^32–37^ In traditional wet-chemical synthesis of metal oxides NCs, an aqueous system has been commonly used, against limited exceptions, such as maghemite^38^ and magnetite,^39^ Mn-based spinels,^40–42^ and In_2_O_3_,^43,44^ which have been produced using hot organic solutions as the reaction media. Nonaqueous colloidal synthesis offers advantages in the precise control of size, composition, morphology, and structure. In recent years, this approach has been increasingly used to prepare NCs of various materials, including metals,^45,46^ alloys,^47–49^ semiconductors,^50,51^ metal oxides (*e.g.*, spinels),^52^ and halide perovskites,^53,54^ with high precision in size, morphology, and purity. Well-controlled SnO_2_ NCs have demonstrated unique physical and chemical properties. For instance, 2 nm SnO_2_ NCs exhibit a strong quantum confinement effect and are ferromagnetic in nature, while 3 nm SnO_2_ NCs show diamagnetic behavior similar to that of bulk SnO_2_.^55^ Moreover, the electronic structure and oxygen vacancies of SnO_2_ NCs can be tailored by doping with a second metal, including rare earth elements (*e.g.*, Tb, Eu, Ce) or other metals (*e.g.*, Zr, Co, Ni, Sb),^56–58^ to enhance conductivity, optimize the energy band gap, and improve optical adsorption properties. As a result, the synthesis of high-quality SnO_2_ NCs, particularly with precise structural control *via* nonaqueous colloidal approaches, remains of significant importance.

In this study, we report the synthesis of SnO_2_ NCs using different deliberately designed protocols, including a hot organic solution system composed of oleic acid and oleylamine as capping ligands, 1-octadecene as a nonpolar solvent, and hydrothermal techniques using various solvents. We varied key factors in these syntheses, such as the oxidizing agent (oxygen), reaction temperature, solvent, and reaction time, and explored their impact on the resulting NCs, particularly in terms of the band gap. The structures and optical properties of the as-synthesized SnO_2_ NCs were characterized by using transmission electron microscopy (TEM), X-ray diffraction (XRD), ultraviolet–visible (UV–vis) absorption spectroscopy, and X-ray photoelectron spectroscopy (XPS). The band gaps and electronic structures were further calculated, and a strategy for adjusting the oxygen vacancies was proposed.

## Methods

### Chemicals

Tin bis­(acetylacetonate)­dichloride (Sn­(acac)_2_Cl_2_, 95%) was obtained from Alfa Aesar. Oleic acid (90%), oleylamine (70%), and 1-octadecene (90%) were sourced from Sigma-Aldrich. Anhydrous ethanol (200 proof), anhydrous hexane (98.5%), and acetone (99.6%) were purchased from Pharmco-AAPER, Merck KGaA (formerly British Drug Houses), and Fisher Scientific, respectively. All chemicals were used as received without further purification.

### Synthetic Procedure

SnO_2_ NCs were synthesized using hot organic solution and hydrothermal approaches. In a typical hot-solution synthesis, 0.40 mmol of tin bis­(acetylacetonate)­dichloride, 0.55 mL of oleylamine, and 0.60 mL of oleic acid were mixed with 7.0 mL of 1-octadecene in a three-neck flask equipped with a condenser. After degassing at 100 °C for 1 h, the temperature of the system was gradually increased to 270 °C at a rate of 10 °C/min under magnetic agitation and argon protection. Upon reaching 270 °C, air was injected into the system at a rate of 36 mL/min for 1 h. The heating source was then removed, and the system was allowed to cool to room temperature naturally. The resulting NCs were isolated by adding excess ethanol and centrifugation. After two washing cycles with a hexane–ethanol mixture and centrifugation, the precipitate was redispersed in hexane and stored under ambient conditions for further use. The suspension appeared black and was labeled as “SnO_2__black”.

A hydrothermal method using autoclaves was employed to prepare two additional types of SnO_2_ samples. In a typical procedure, 0.40 mmol of tin bis­(acetylacetonate)­dichloride, 0.55 mL of oleylamine, 0.60 mL of oleic acid, and 7.0 mL of 1-octadecene were loaded in a Teflon-lined stainless-steel autoclave and heated in a preheated oven at 200 °C for 24 h. After being cooled to room temperature, the product was collected and purified by adding sufficient ethanol followed by centrifugation. After the washing procedure was repeated, the final product was redispersed in hexane as a stock suspension. This sample appeared grey and was labeled as “SnO_2__grey”. The third type of sample was prepared using the same hydrothermal method, with the same recipe and procedure but by replacing 1-octadecene with anhydrous ethanol. The suspension appeared white, and this sample was labeled as “SnO_2__white”.

### Characterizations

XRD patterns were recorded using a PANalytical X’Pert powder X-ray diffractometer equipped with a Cu Kα1 radiation source. Low-resolution TEM images were captured using an FEI Tecnai Spirit TEM operating at 120 kV and an FEI Tecnai G2 F20 ST TEM working at 200 kV. For high-resolution TEM (HRTEM) imaging, an FEI Titan 80-300 TEM equipped with a field emission gun and a CEOS image corrector was used. UV–vis absorption spectroscopy measurements were performed on a VARIAN Cary 50-Bio UV–vis spectrophotometer. XPS analysis was conducted on a PHI 5000 VersaProbe system (Physical Electronics, Inc.), with binding energies corrected for specimen charging by referencing the C 1s peak to 284.6 eV.

## Results and Discussion

In the synthesis of SnO_2_ NCs, oleylamine, in combination with oleic acid, provides an alkalescent environment and surface capping ligands that facilitate nucleation and particle growth, while 1-octadecene serves as a nonpolar solvent. It is believed that under these reaction conditions, tin­(IV) species are further converted into SnO_2_ during the aging process^34,52^ by the introduction of additional oxygen from the air, as depicted in the schematic diagram in Figure S1. Figure 1 shows low-magnification TEM and high-resolution TEM (HRTEM) images of the as-synthesized SnO_2_ NCs, with size distribution histograms for the three samples presented in Figures S2–S4. The SnO_2_ NCs from the hot organic solution synthesis (SnO_2__black), collected under ambient pressure, exhibit a spherical morphology with an average diameter of 4.2 ± 0.8 nm (Figure S2). When the temperature was lowered to 200 °C and the reaction time was extended to 24 h in an autoclave, larger SnO_2_ NCs were produced, maintaining a spherical morphology, with an average diameter of 6.3 ± 1.2 nm (Figure S3). The formation of NCs occurs in two stages: nucleation (seed formation) and crystal growth. It is believed that the higher synthesis temperature and abundant oxygen supply (*via* air injection at 36 mL/min) for the SnO_2__black sample result in a faster nucleation rate, leading to a higher concentration of SnO_2_ seeds. Consequently, the concentration of remaining Sn-precursors is lower during the crystal growth stage, resulting in a smaller particle size (4.2 nm, Figure 1a and Figure S2) compared to that of SnO_2__grey (6.3 nm, Figure 1b and Figure S3), where the lower temperature and high concentration of the Sn-precursor during the growth stage lead to slower nucleation and larger crystal size, respectively. The introduction of a small amount of ethanol in place of an equal volume of 1-octadecene yields the SnO_2__white sample, which exhibits a rod-like morphology (Figure 1c). The average length of these rods was 9.6 ± 1.9 nm with a width of 3.9 ± 0.6 nm (Figure S4). In this case, ethanol acts as a polar solvent and a capping ligand on specific crystal planes, promoting epitaxial growth and the formation of rod-like SnO_2_ NCs.^10^ The lattice spacing observed in the representative HRTEM images for all three samples was consistent with 0.33 nm, corresponding to the (110) planes of the cassiterite SnO_2_ crystal structure.

**Figure 1:**
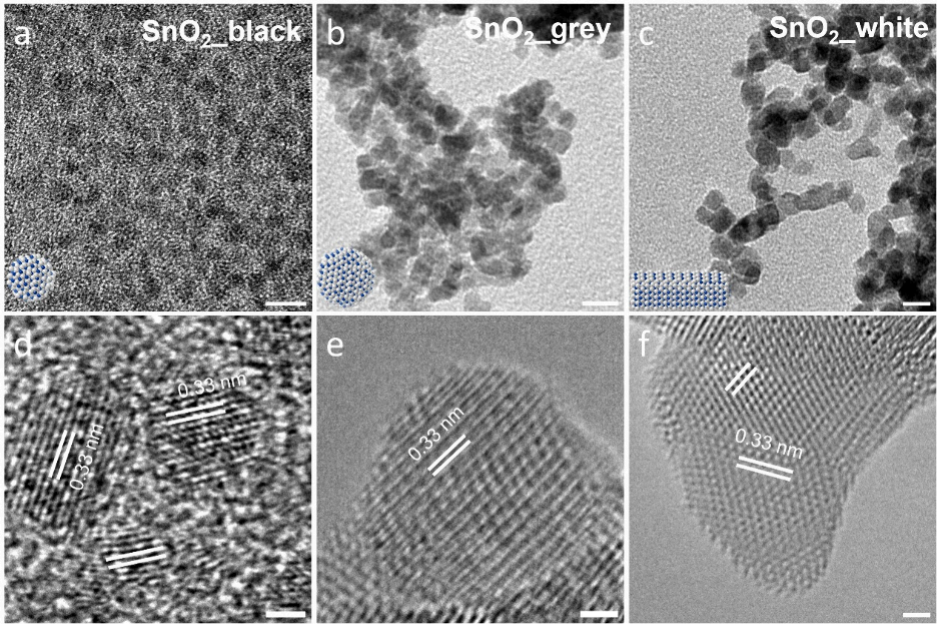
TEM and HRTEM images of SnO_2_ nanocrystals. (a–c) TEM images of (a) SnO_2__black, (b) SnO_2__grey, and (c) SnO_2__white; (d–f) typical HRTEM images of (d) SnO_2__black, (e) SnO_2__grey, and (f) SnO_2__white. Scale bars in TEM and HRTEM panels are 10 and 2 nm, respectively.

To further confirm the crystallographic structures of the SnO_2_ NCs, XRD patterns of the three as-prepared samples were recorded and are shown in Figure 2a. All diffraction peaks in the XRD patterns can be clearly indexed to the tetragonal cassiterite structure of SnO_2_ (JCPDS no. 41-1445), with no additional impurity phases detected, indicating the high purity of the obtained products. The average crystallite sizes for SnO_2__black, SnO_2__grey, and SnO_2__white were estimated to be 4.4, 6.0, and 8.6 nm, respectively, using the Scherrer formula based on the (110) reflection. These values are consistent with the crystallite sizes obtained from the TEM images. Additionally, Pawley fitting analysis further supports the *P*4_2_/*mnm* symmetry for all three samples, and the calculated unit cell parameters confirm the cassiterite crystal structure (Figure S5).

**Figure 2:**
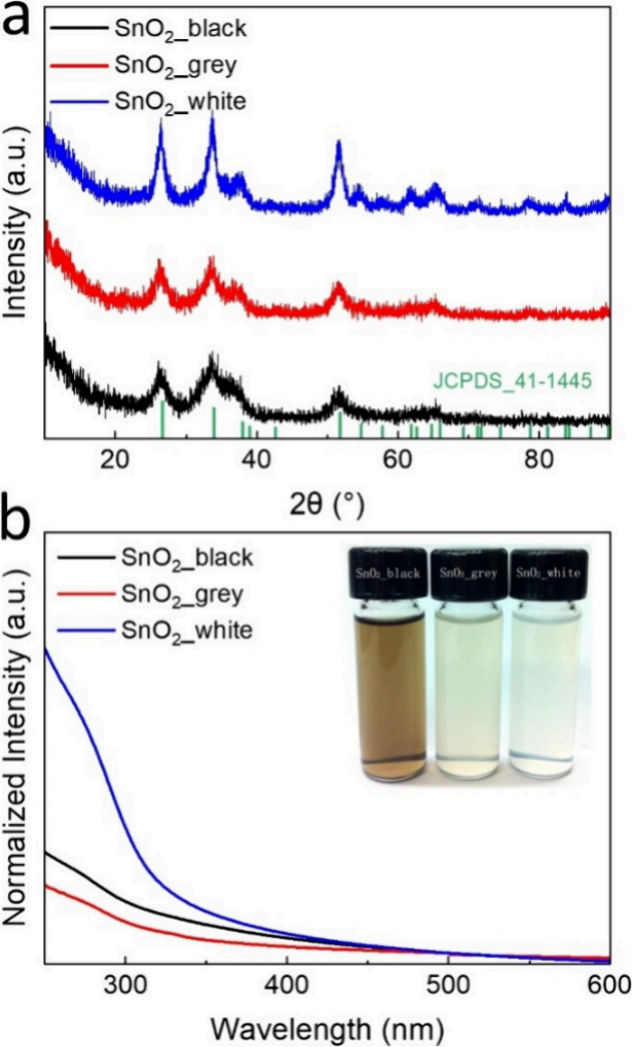
XRD patterns (a) and UV–visible absorption spectra (b) of the three samples. The inset in (b) shows a photograph of SnO_2_ nanocrystals suspended in hexane following purification.

We measured the UV–vis absorption spectra of the three SnO_2_ samples to investigate the optical properties. As shown in Figure 2b, the absorption onset for all samples occurred in the range of 320 to 300 nm. Compared to SnO_2__black, the absorption onset of SnO_2__grey is red-shifted due to particle growth. The band gaps of these SnO_2_ NCs were estimated using the classical Tauc plotting method.^59^ This approach assumes that the energy-dependent absorption coefficient, α, can be expressed by eqs 1 and 2, shown as follows:

$${\left(\alpha hv\right)}^{1/\gamma }=B\left(hv-E_{g}\right)$$ 1

2 v=c/λ

where *h* is the Planck constant (6.626 × 10^–34^ m^2^·kg/s), *v* is the frequency of the photon, *c* is the speed of light (3.0 × 10^8^ m/s in free space), λ is the wavelength of light (in meters), *E*_g_ is the band gap energy (in eV), and *B* is a constant. The γ factor depends on the type of electron transition, with a value of 2 for indirect band gaps and a value of 1/2 for direct band gaps.

It is well-known that γ = 1/2 (for allowed direct transitions) provides the best description of absorption measurements.^35,36^ Figure 3 shows a representative plot of (*αhν*)^2^ versus *hν* for a direct transition. The extrapolated value of (*αhν*)^2^ = 0 to the abscissa yields the absorption edge energy, corresponding to band gap energies of 3.95, 3.92, and 4.05 eV for SnO_2__black, SnO_2__grey, and SnO_2__white, respectively. In agreement with the small crystallite sizes observed with TEM for all three types of SnO_2_ NCs, these values indicate a significant blue shift compared to the band gap energy of bulk tetragonal SnO_2_ (*E*_*g*_ = 3.60 eV, with a Bohr radius of 2.7 nm), due to quantum confinement effects.^55^ Interestingly, despite having different size distributions, the SnO_2__black and SnO_2__grey NCs exhibited similar band gap values. This suggests that the different synthetic protocols resulted in NCs with varying electronic structures and properties. Additionally, it is worth noting that the band gap of SnO_2__white is larger than those of SnO_2__black and SnO_2__grey, likely due to the smaller width (3.9 nm) of the rod-shaped SnO_2_ NCs, in comparison to the spherical SnO_2__black (4.2 nm) and SnO_2__grey (6.3 nm).

**Figure 3:**
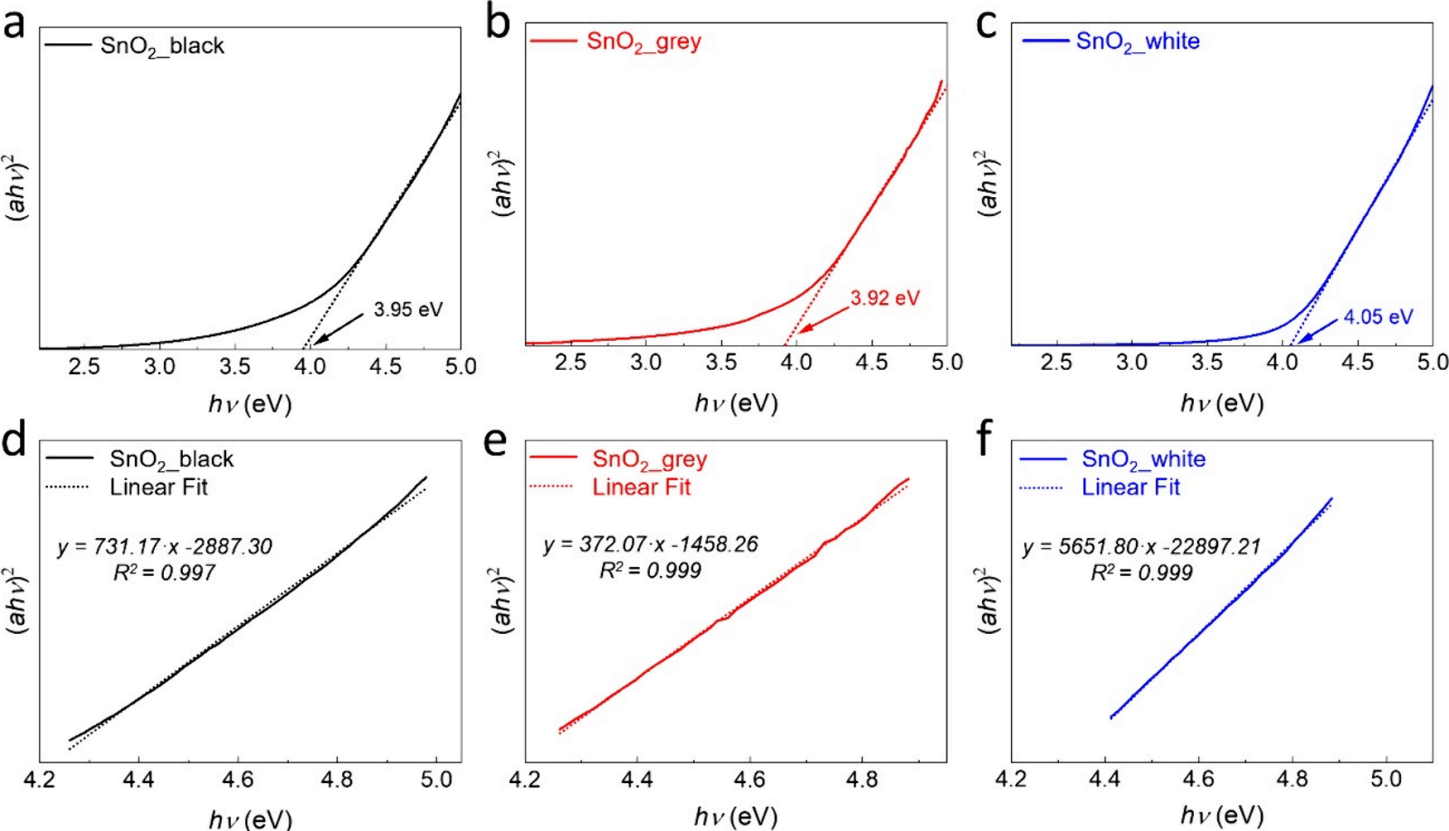
Tauc plots derived from UV–vis spectra of the as-synthesized samples: (a) SnO_2__black (black line), (b) SnO_2__grey (red line), and (c) SnO_2__white (blue line). Extrapolating the straight linear region (4.3 to 4.9 eV) in (d) SnO_2__black, (e) SnO_2__grey, and (f) SnO_2__white to the abscissa yields the value of the band gap (*E*_g_) for each sample, respectively.

X-ray photoelectron spectroscopy (XPS) was employed to characterize the binding energies and oxidation states of the elements in the fresh powders, as shown in Figure 4 and Figure S6. The Sn 3d spectra in Figure 4a display symmetrical peaks, with the binding energy of Sn 3d_5/2_ peaking at 486.6, 486.7, and 486.9 eV for the three SnO_2_ NCs, respectively. These values are attributed to Sn^4+^,^17^ and no Sn^2+^ signals were detected from the Sn 3d peak for any of the three samples, which is consistent with the XRD results, confirming the presence of pure SnO_2_ in the tetragonal cassiterite form. As shown in Figure 4b, the O 1s spectra of the SnO_2_ NC samples, ranging from 530 to 534 eV, were deconvoluted using a multi-Gaussian function into two components for all three samples. Specifically, the lower binding energy peak at 530.7 eV corresponds to oxygen coordinated with tin, while the higher binding energy component centered at 531.95 eV arises due to oxygen loss, indicating nonstoichiometric defects in SnO_2_ NCs.^60^ The higher proportion of the peak at 531.95 eV in SnO_2__black suggests a greater concentration of oxygen defects compared to the other two samples, which contributes to the observed band gap shift.

**Figure 4:**
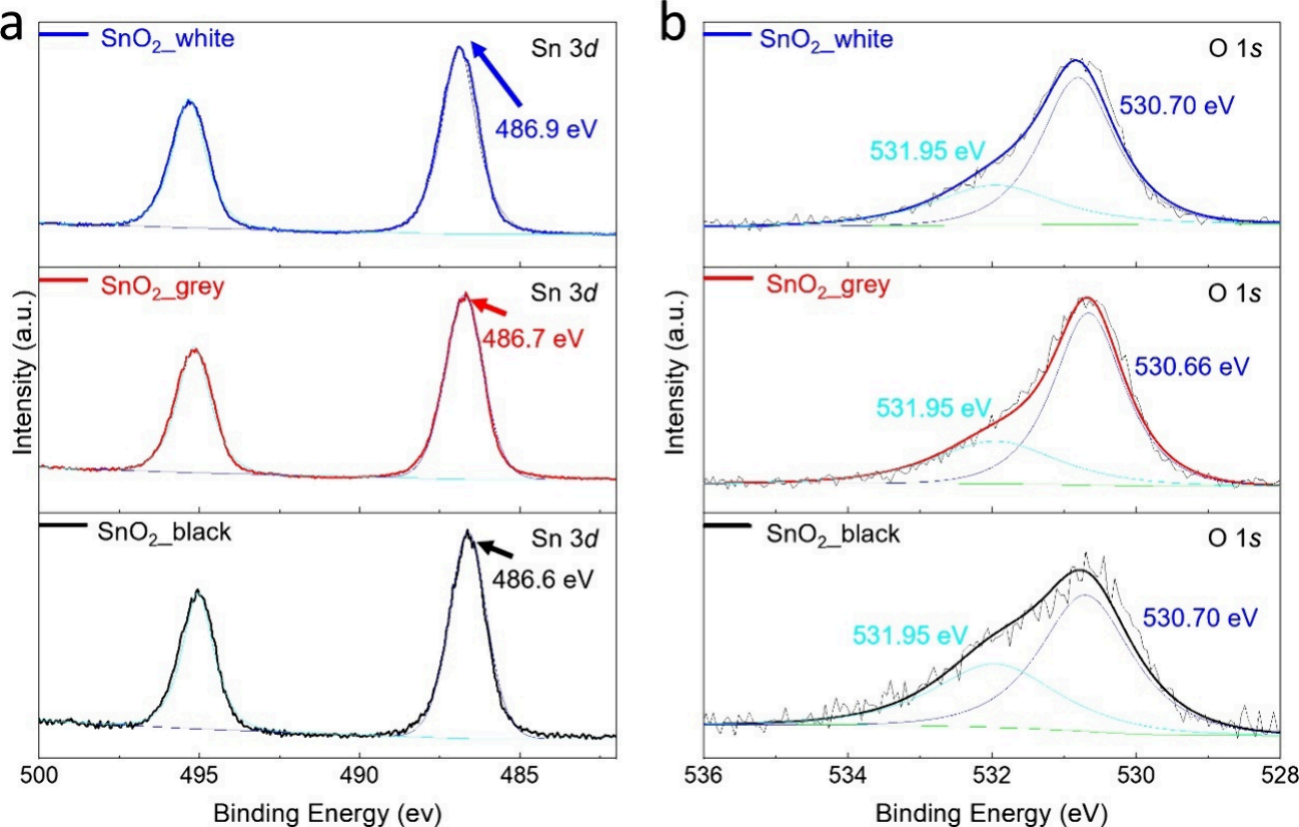
XPS spectra of SnO_2__black, SnO_2__grey, and SnO_2__white: (a) Sn 3d and (b) deconvoluted O 1s.

To gain further insights into the difference in their electronic properties, we performed valence band (VB) XPS characterization near the Fermi level (*E*_f_) to investigate the electronic structure of the as-synthesized SnO_2_ NCs (Figure 5).^61^ The peak in the VB energy region is located between 3 and 13 eV relative to *E*_f_. All three samples exhibit multiple peaks in the VB region. The VB maximum energy was determined by extrapolating the linear region (from 4.4 to 3.5 eV) to the baseline (Figure S7). Assuming the *E*_f_ for an n-type semiconductor, the VB edge of the maximum energy was measured to be 3.11, 3.48, and 3.51 eV for SnO_2__black, SnO_2__grey, and SnO_2__white, respectively, as shown in Figure 5a–c.

**Figure 5:**
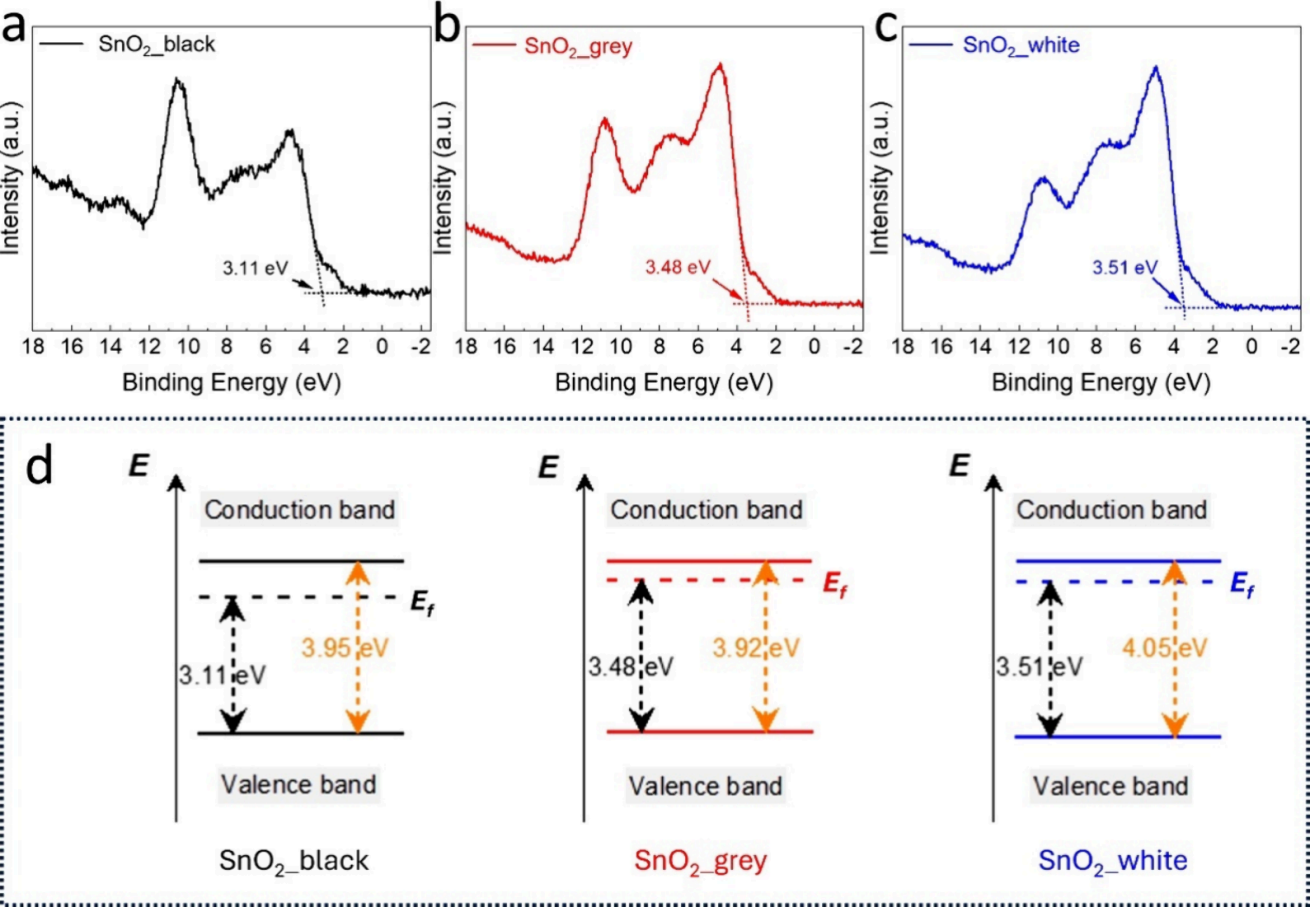
Valence band XPS spectra of the three samples (a) SnO_2__black, (b) SnO_2__grey, and (c) SnO_2__white. (d) The valence band energy (*E*_V_) and conduction band energy (*E*_C_) relative to the Fermi level (*E*_f_) in the band structures of the three SnO_2_ NC samples.

Combined with the band gaps (Figure 3) from the optical absorption spectra, the conduction band minima of SnO_2__black, SnO_2__grey, and SnO_2__white are estimated to occur at approximately −0.84, −0.44, and −0.54 eV, respectively. A schematic illustration of the band gap structure relative to *E*_f_ for the three types of SnO_2_ NCs is shown in Figure 5d. Compared to the other two types of SnO_2_ samples synthesized hydrothermally, SnO_2__black, produced from the hot organic solution, exhibits the smallest VB maximum energy, suggesting that the upward shift of the VB maximum contributes to a narrower band gap. This results in a similar band gap value derived from the optical absorption spectra for SnO_2__black and SnO_2__grey, despite SnO_2__black having a smaller diameter. The abundant oxygen defects in SnO_2__black, as confirmed by the O 1s XPS analysis, help explain the observed narrower band gap and VB shift. These characteristics can be attributed to the high concentration of oxygen vacancies within the SnO_2__black sample, which were formed in the colloidal system through air injection during the relatively short synthesis period. This result aligns with previous experimental studies and first-principles calculations based on density functional theory (DFT).^60^

## Conclusions

In this study, we successfully synthesized highly crystalline SnO_2_ NCs with diverse sizes and morphologies by using different synthesis approaches. We identified key factors that influence the synthesis process and demonstrated an effective strategy for controlling the size and shape by adjusting nucleation duration and growth rates. Specifically, the hot organic solution method promoted the formation of smaller SnO_2_ NCs with abundant oxygen defects, while extending the reaction time or replacing the nonpolar solvent with ethanol in the lower-temperature hydrothermal process resulted in larger spherical or rod-like SnO_2_ NCs with fewer oxygen vacancies. The band gaps and electronic structures of these SnO_2_ samples were characterized using optical absorption spectra and valence band XPS. The results showed that the hot-solution-derived SnO_2_ NCs exhibited a narrower band gap, an upshifted valence band, and a higher concentration of oxygen vacancies compared to those synthesized hydrothermally. These variations in band gaps and electronic structures suggest that SnO_2_ NCs can be tailored for a variety of applications. The strategies presented in this work illustrate the potential for deliberately designing NCs by understanding their formation mechanisms.

## Supplementary Material



## Notes

Schematic of the synthesis of SnO_2_ nanocrystal samples, size distribution histograms of SnO_2_ samples, lattice parameters of SnO_2_ samples obtained from Pawley fitting, XPS spectra of C 1s for the three SnO_2_ samples, and extrapolation of the straight linear regions (3.9 to 3.5 eV) to the baseline for the three samples, yielding the maximum energy of the valence band (*E*_V_) (PDF)

## #

College of Chemistry, Beijing University of Chemical Technology, Beijing 100029, China

## References

[1] Ahamed M., Akhtar M. J., Khan M. A. M., Alhadlaq H. A. (2021). SnO_2_-Doped
ZnO/Reduced Graphene Oxide Nanocomposites: Synthesis, Characterization,
and Improved Anticancer Activity via Oxidative Stress Pathway. Int. J. Nanomedicine.

[2] Wang Z., Gao S., Fei T., Liu S., Zhang T. (2019). Construction of ZnO/SnO_2_ Heterostructure on Reduced Graphene Oxide for Enhanced Nitrogen
Dioxide Sensitive Performances at Room Temperature. ACS Sensors.

[3] Chávez-Calderón A., Paraguay-Delgado F., Orrantia-Borunda E., Luna-Velasco A. (2016). Size Effect
of SnO_2_ Nanoparticles on Bacteria Toxicity and Their Membrane
Damage. Chemosphere.

[4] Wang Y., Ding J., Liu Y., Liu Y., Cai Q., Zhang J. (2015). SnO_2_@Reduced Graphene
Oxide Composite for High Performance
Lithium-ion Battery. Ceram. Int..

[5] Lin J., Peng Z., Xiang C., Ruan G., Yan Z., Natelson D., Tour J. M. (2013). Graphene Nanoribbon and Nanostructured
SnO_2_ Composite Anodes for Lithium Ion Batteries. ACS Nano.

[6] Reddy M. V., Subba Rao G. V., Chowdari B. V. R. (2013). Metal Oxides and Oxysalts as Anode
Materials for Li Ion Batteries. Chem. Rev..

[7] Meduri P., Pendyala C., Kumar V., Sumanasekera G. U., Sunkara M. K. (2009). Hybrid Tin Oxide Nanowires as Stable
and High Capacity
Anodes for Li-Ion Batteries. Nano Lett..

[8] Garcia
Romero D., Di Mario L., Yan F., Ibarra-Barreno C. M., Mutalik S., Protesescu L., Rudolf P., Loi M. A. (2024). Understanding
the Surface Chemistry of SnO_2_ Nanoparticles for High Performance
and Stable Organic Solar Cells. Adv. Funct.
Mater..

[9] Di
Mario L., Garcia Romero D., Wang H., Tekelenburg E. K., Meems S., Zaharia T., Portale G., Loi M. A. (2024). Outstanding
Fill Factor in Inverted Organic Solar Cells with SnO_2_ by
Atomic Layer Deposition. Adv. Mater..

[10] Peiris T. A. N., Weerasinghe H. C., Sharma M., Kim J.-E., Michalska M., Chandrasekaran N., Senevirathna D. C., Li H., Chesman A. S. R., Vak D., Jasieniak J. J. (2022). Non-Aqueous
One-Pot SnO_2_ Nanoparticle Inks and Their Use in Printable
Perovskite Solar Cells. Chem. Mater..

[11] Park S. Y., Zhu K. (2022). Advances in SnO_2_ for Efficient and Stable *n-i-p* Perovskite Solar Cells. Adv. Mater..

[12] Xiong L., Li J., Ye F., Wang H., Guo Y., Ming X., Chen Q., Zhang S., Xie R., Chen Z., Lv Y., Hu G., He Y., Fang G. (2021). Bifunctional SnO_2_ Colloid
Offers No Annealing Effect Compact Layer and Mesoporous
Scaffold for Efficient Perovskite Solar Cells. Adv. Funct. Mater..

[13] Chen X., Liu L., Yu P. Y., Mao S. S. (2011). Increasing Solar Absorption for Photocatalysis
with Black Hydrogenated Titanium Dioxide Nanocrystals. Science.

[14] Chen Z., Huang Q., Zhang Y., Sheng P., Cui Z. (2021). Confined Generation
of Homogeneously Dispersed Au and SnO_2_ Nanoparticles in
Layered Silicate as Synergistic Catalysts. Langmuir.

[15] Lang R., Du X., Huang Y., Jiang X., Zhang Q., Guo Y., Liu K., Qiao B., Wang A., Zhang T. (2020). Single-Atom Catalysts
Based on the Metal-Oxide Interaction. Chem.
Rev..

[16] Luque P. A., Garrafa-Gálvez H. E., Nava O., Olivas A., Martínez-Rosas M. E., Vilchis-Nestor A. R., Villegas-Fuentes A., Chinchillas-Chinchillas M. J. (2021). Efficient Sunlight
and UV Photocatalytic Degradation of Methyl Orange, Methylene Blue
and Rhodamine B, Using Citrus × paradisi Synthesized SnO_2_ Semiconductor Nanoparticles. Ceram.
Int..

[17] Fan C.-M., Peng Y., Zhu Q., Lin L., Wang R.-X., Xu A.-W. (2013). Synproportionation Reaction for the
Fabrication of Sn^2+^ Self-Doped SnO_2‑x_ Nanocrystals with Tunable Band
Structure and Highly Efficient Visible Light Photocatalytic Activity. J. Phys. Chem. C.

[18] Jang J.-S., Choi S.-J., Kim S.-J., Hakim M., Kim I.-D. (2016). Rational
Design of Highly Porous SnO_2_ Nanotubes Functionalized with
Biomimetic Nanocatalysts for Direct Observation of Simulated Diabetes. Adv. Funct. Mater..

[19] Jang B.-H., Landau O., Choi S.-J., Shin J., Rothschild A., Kim I.-D. (2013). Selectivity Enhancement
of SnO_2_ Nanofiber
Gas Sensors by Functionalization with Pt Nanocatalysts and Manipulation
of the Operation Temperature. Sens. Actuators
B Chem..

[20] Sano K., Ishida T., Shimada T., Tachibana H., Takashima M., Ohtani B., Takagi S., Inoue H. (2024). Photocatalytic
Hydrogen Evolution from a Transparent Aqueous Dispersion of Quantum-Sized
SnO_2_ Nanoparticles: Effect of Electron Trap Density within
One Particle. J. Phys. Chem. C.

[21] Shabna S., Dhas S. S. J., Biju C. S. (2023). Potential Progress in SnO_2_ Nanostructures for Enhancing Photocatalytic Degradation of Organic
Pollutants. Catal. Commun..

[22] Chen M., Chen X., Ma W., Sun X., Wu L., Lin X., Yang Y., Li R., Shen D., Chen Y., Chen S. (2022). Highly Stable SnO_2_-Based
Quantum-Dot Light-Emitting Diodes
with the Conventional Device Structure. ACS
Nano.

[23] Rohilla D., Chaudhary S., Umar A. (2021). An Overview of Advanced Nanomaterials
for Sensor Applications. Eng. Sci..

[24] Sun Y., Lei F., Gao S., Pan B., Zhou J., Xie Y. (2013). Atomically
Thin Tin Dioxide Sheets for Efficient Catalytic Oxidation of Carbon
Monoxide. Angew. Chem., Int. Ed..

[25] Gyger F., Hübner M., Feldmann C., Barsan N., Weimar U. (2010). Nanoscale
SnO_2_ Hollow Spheres and Their Application as a Gas-Sensing
Material. Chem. Mater..

[26] Lu G., Ocola L. E., Chen J. (2009). Room-Temperature
Gas Sensing Based
on Electron Transfer between Discrete Tin Oxide Nanocrystals and Multiwalled
Carbon Nanotubes. Adv. Mater..

[27] Wang Y., Jiang X., Xia Y. (2003). A Solution-Phase, Precursor Route
to Polycrystalline SnO_2_ Nanowires That Can Be Used for
Gas Sensing under Ambient Conditions. J. Am.
Chem. Soc..

[28] Yun H.-S., Seo Y.-H., Seo C.-E., Kim H. S., Yoo S. B., Kang B. J., Jeon N. J., Jung E. H. (2024). Surface Engineering
of Tin Oxide Nanoparticles by pH Modulation Facilitates Homogeneous
Film Formation for Efficient Perovskite Solar Modules. Adv. Energy Mater..

[29] Paik M. J., Kim Y. Y., Kim J., Park J., Seok S. I. (2024). Ultrafine
SnO_2_ Colloids with Enhanced Interface Quality for High-Efficiency
Perovskite Solar Cells. Joule.

[30] Noh Y. W., Lee J. H., Jin I. S., Park S. H., Jung J. W. (2019). Tailored
Electronic Properties of Zr-Doped SnO_2_ Nanoparticles for
Efficient Planar Perovskite Solar Cells with Marginal Hysteresis. Nano Energy.

[31] Bai Y., Fang Y., Deng Y., Wang Q., Zhao J., Zheng X., Zhang Y., Huang J. (2016). Low Temperature Solution-Processed
Sb:SnO_2_ Nanocrystals for Efficient Planar Perovskite Solar
Cells. ChemSusChem.

[32] Matussin S., Harunsani M. H., Tan A. L., Khan M. M. (2020). Plant-Extract-Mediated
SnO_2_ Nanoparticles: Synthesis and Applications. ACS Sustainable Chem. Eng..

[33] Haritha E., Roopan S. M., Madhavi G., Elango G., Al-Dhabi N. A., Arasu M. V. (2016). Green Chemical Approach
towards the Synthesis of SnO_2_ NPs in Argument with Photocatalytic
Degradation of Diazo
Dye and its Kinetic Studies. J. Photochem. Photobiol.,
B.

[34] Zhang L., Ge S., Zuo Y., Zhang B., Xi L. (2010). Influence of Oxygen
Flow Rate on the Morphology and Magnetism of SnO_2_ Nanostructures. J. Phys. Chem. C.

[35] Liu B., Zeng H. C. (2004). Salt-Assisted Deposition
of SnO_2_ on α-MoO_3_ Nanorods and Fabrication
of Polycrystalline SnO_2_ Nanotubes. J. Phys. Chem. B.

[36] Gu F., Wang S. F., Lü M. K., Zhou G. J., Xu D., Yuan D. R. (2004). Photoluminescence
Properties of SnO_2_ Nanoparticles
Synthesized by Sol-Gel Method. J. Phys. Chem.
B.

[37] Ye Y., Wang P., Dai E., Liu J., Tian Z., Liang C., Shao G. (2014). A Novel Reduction Approach to Fabricate
Quantum-Sized SnO_2_-Conjugated Reduced Graphene Oxide Nanocomposites
as Non-enzymatic Glucose Sensors. Phys. Chem.
Chem. Phys..

[38] Hyeon T., Lee S. S., Park J., Chung Y., Na H. B. (2001). Synthesis
of Highly Crystalline and Monodisperse Maghemite Nanocrystallites
without a Size-Selection Process. J. Am. Chem.
Soc..

[39] Caruntu D., Caruntu G., Chen Y., O’Connor C. J., Goloverda G., Kolesnichenko V. L. (2004). Synthesis of Variable-Sized Nanocrystals
of Fe_3_O_4_ with High Surface Reactivity. Chem. Mater..

[40] Zhou M., Yoon D., Yang Y., Zhang L., Li C., Wang H., Sharma A., Jiang S., Muller D. A., Abruña H. D., Fang J. (2023). Enhanced Oxygen Reduction Performance
on {101} CoMn_2_O_4_ Spinel Facets. ACS Energy Lett..

[41] Sun Y., Liao H., Wang J., Chen B., Sun S., Ong S. J. H., Xi S., Diao C., Du Y., Wang J.-O., Breese M. B. H., Li S., Zhang H., Xu Z. J. (2020). Covalency Competition
Dominates the Water Oxidation Structure-Activity
Relationship on Spinel Oxides. Nat. Catal..

[42] Yang Y., Xiong Y., Holtz M. E., Feng X., Zeng R., Chen G., DiSalvo F. J., Muller D. A., Abruña H. D. (2019). Octahedral
Spinel Electrocatalysts for Alkaline Fuel Cells. Proc. Natl. Acad. Sci. U. S. A..

[43] Jiang S., Chen X., Huang X., Li C., Wang Z., Zhao B., Zhang L., Zhou G., Fang J. (2024). Randomly Layered
Superstructure of In_2_O_3_ Truncated Nano-Octahedra
and Its High-Pressure Behavior. J. Am. Chem.
Soc..

[44] Liu Q., Lu W., Ma A., Tang J., Lin J., Fang J. (2005). Study of Quasi-Monodisperse
In_2_O_3_ Nanocrystals: Synthesis and Optical Determination. J. Am. Chem. Soc..

[45] Ma Z., Mohapatra J., Wei K., Liu J. P., Sun S. (2023). Magnetic Nanoparticles:
Synthesis, Anisotropy, and Applications. Chem.
Rev..

[46] Shi Y., Lyu Z., Zhao M., Chen R., Nguyen Q. N., Xia Y. (2021). Noble-Metal
Nanocrystals with Controlled Shapes for Catalytic and Electrocatalytic
Applications. Chem. Rev..

[47] Zhou M., Li C., Fang J. (2021). Noble-Metal
Based Random Alloy and Intermetallic Nanocrystals:
Syntheses and Applications. Chem. Rev..

[48] Gilroy K. D., Ruditskiy A., Peng H.-C., Qin D., Xia Y. (2016). Bimetallic
Nanocrystals: Syntheses, Properties, and Applications. Chem. Rev..

[49] Chen M., Kim J., Liu J. P., Fan H., Sun S. (2006). Synthesis of FePt Nanocubes
and Their Oriented Self-Assembly. J. Am. Chem.
Soc..

[50] Lu W., Fang J., Stokes K. L., Lin J. (2004). Shape Evolution and
Self Assembly of Monodisperse PbTe Nanocrystals. J. Am. Chem. Soc..

[51] Cho K.-S., Talapin D. V., Gaschler W., Murray C. B. (2005). Designing PbSe Nanowires
and Nanorings through Oriented Attachment of Nanoparticles. J. Am. Chem. Soc..

[52] Zhou M., Wang H., Zhang L., Li C., Kumbhar A., Abruña H. D., Fang J. (2022). Facet Impact of CuMn_2_O_4_ Spinel Nanocatalysts on Enhancement of the Oxygen
Reduction
Reaction in Alkaline Media. ACS Catal..

[53] Holder C. F., Fanghanel J., Xiong Y., Dabo I., Schaak R. E. (2020). Phase-Selective
Solution Synthesis of Perovskite-Related Cesium Cadmium Chloride Nanoparticles. Inorg. Chem..

[54] Yin T., Liu B., Yan J., Fang Y., Chen M., Chong W. K., Jiang S., Kuo J.-L., Fang J., Liang P., Wei S., Loh K. P., Sum T. C., White T. J., Shen Z. X. (2019). Pressure-Engineered
Structural and Optical Properties of Two-Dimensional (C_4_H_9_NH_3_)_2_PbI_4_ Perovskite
Exfoliated nm-Thin Flakes. J. Am. Chem. Soc..

[55] Dutta D., Bahadur D. (2012). Influence of Confinement Regimes on Magnetic Property
of Pristine SnO_2_ Quantum Dots. J.
Mater. Chem..

[56] Kandasamy M., Seetharaman A., Sivasubramanian D., Nithya A., Jothivenkatachalam K., Maheswari N., Gopalan M., Dillibabu S., Eftekhari A. (2018). Ni-Doped SnO_2_ Nanoparticles for Sensing
and Photocatalysis. ACS Appl. Nano Mater..

[57] Nasir Z., Shakir M., Wahab R., Shoeb M., Alam P., Khan R. H., Mobin M., Lutfullah (2017). Co-precipitation Synthesis and Characterization
of Co-doped SnO_2_ NPs, HSA Interaction *via* Various Spectroscopic Techniques and Their Antimicrobial and Photocatalytic
Activities. Int. J. Biol. Macromol..

[58] Wang Y., Djerdj I., Smarsly B., Antonietti M. (2009). Antimony-Doped
SnO_2_ Nanopowders with High Crystallinity for Lithium-Ion
Battery Electrode. Chem. Mater..

[59] Makuła P., Pacia M., Macyk W. (2018). How To Correctly Determine the Band
Gap Energy of Modified Semiconductor Photocatalysts Based on UV-Vis
Spectra. J. Phys. Chem. Lett..

[60] Yang Y., Wang Y., Yin S. (2017). Oxygen Vacancies Confined in SnO_2_ Nanoparticles for Desirable Electronic Structure and Enhanced
Visible Light Photocatalytic Activity. Appl.
Surf. Sci..

[61] Eda Y., Manaka T., Hanawa T., Chen P., Ashida M., Noda K. (2022). X-ray Photoelectron
Spectroscopy-Based Valence Band Spectra of Passive
Films on Titanium. Surf. Interface Anal..

