# Supplementary material for: Controlled Synthesis of SnO2 Nanocrystals with Tunable Band Gaps
Source: Precis Chem. 2025 Mar 17;3(8):463–9. doi: 10.1021/prechem.4c00107 (PMC12381735; doi:10.1021/prechem.4c00107)
Supplement: Supplementary file 1 [file pc4c00107_si_001.pdf]

Supporting Information

Controlled Synthesis of SnO_2 Nanocrystals with Tunable Band Gaps

Can Li,[1,†] Xin Shu,[1,†,§] Jun Zhang,[2] Joseph Delgado,[1] Prabhu Bharathan,[3] Yuxuan Wang,[3] Chenyu Wang,[1] and Jiye Fang*[1,3]

[1]Department of Chemistry, State University of New York at Binghamton, Binghamton, New York 13902, United States

[2]School of Materials Science and Engineering, China University of Petroleum (East China), Qingdao 266580, China

[3]Materials Science and Engineering Program, State University of New York at Binghamton, Binghamton, New York 13902, United States

[†]These authors contributed equally to this work.

Present address:

[§]College of Chemistry, Beijing University of Chemical Technology, Beijing 100029, China

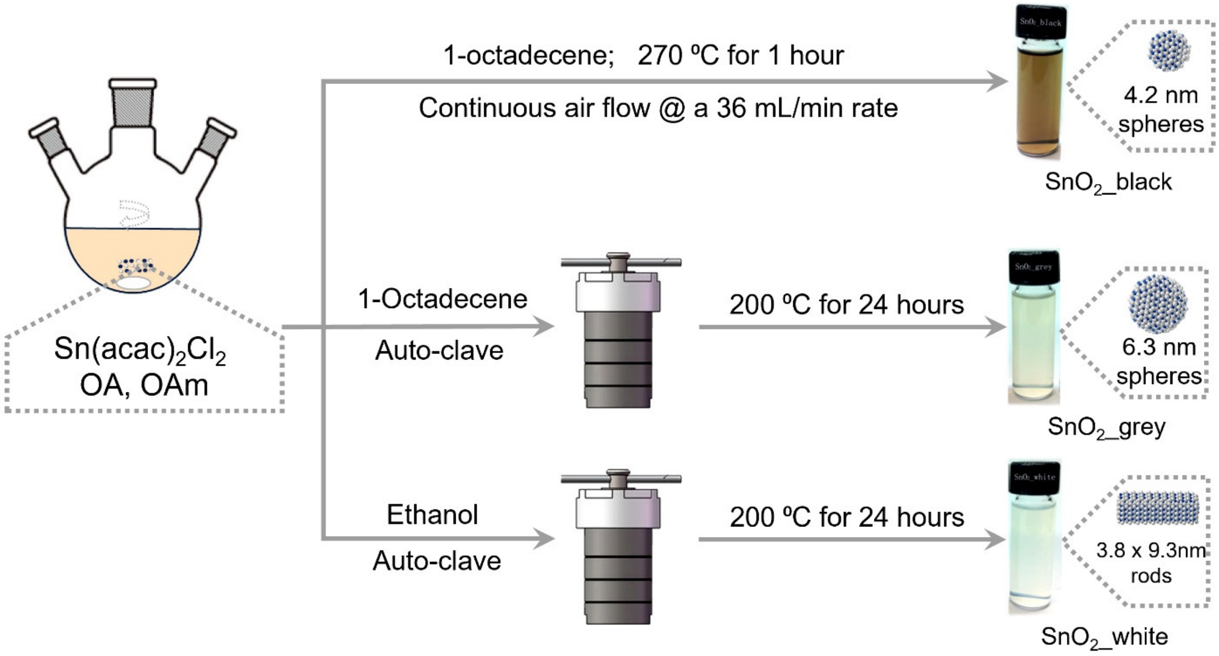

Figure S1. Schematic illustration of the synthesis of SnO_2 nanocrystals with varying sizes, morphologies, and suspension colors (black, grey, and white). $Sn(acac)_2Cl_2$: bis(acetylacetonate)dichloride; OA: oleic acid; OAm: oleylamine.

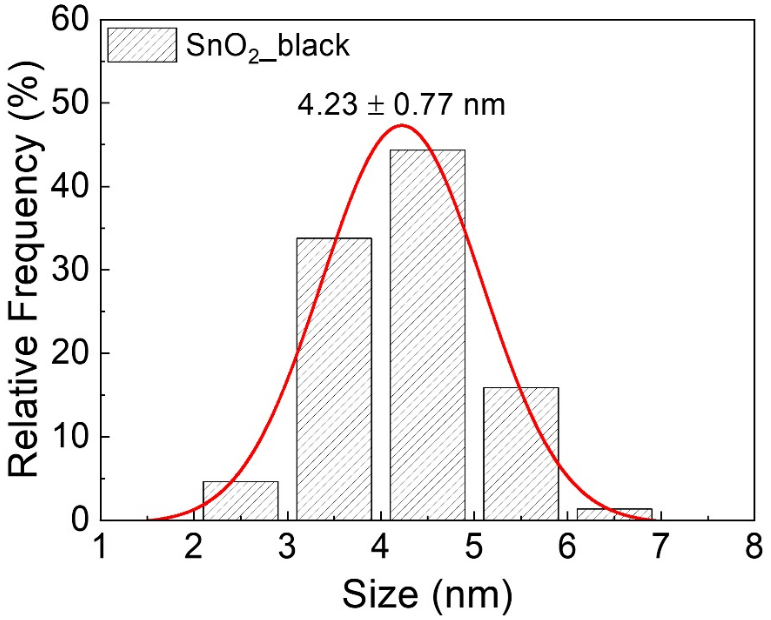

Figure S2. Size distribution histogram of the spherical SnO_2 sample (SnO_2_black).

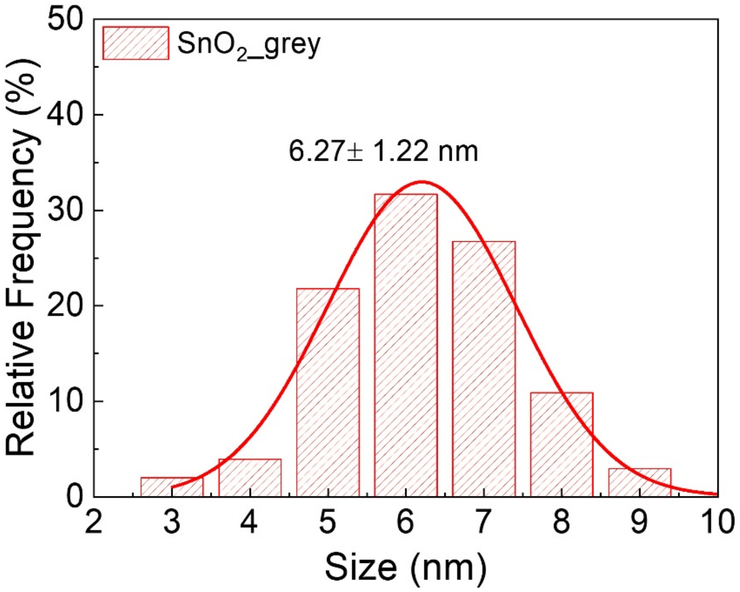

Figure S3. Size distribution histogram of the spherical SnO_2 sample (SnO_2_grey).

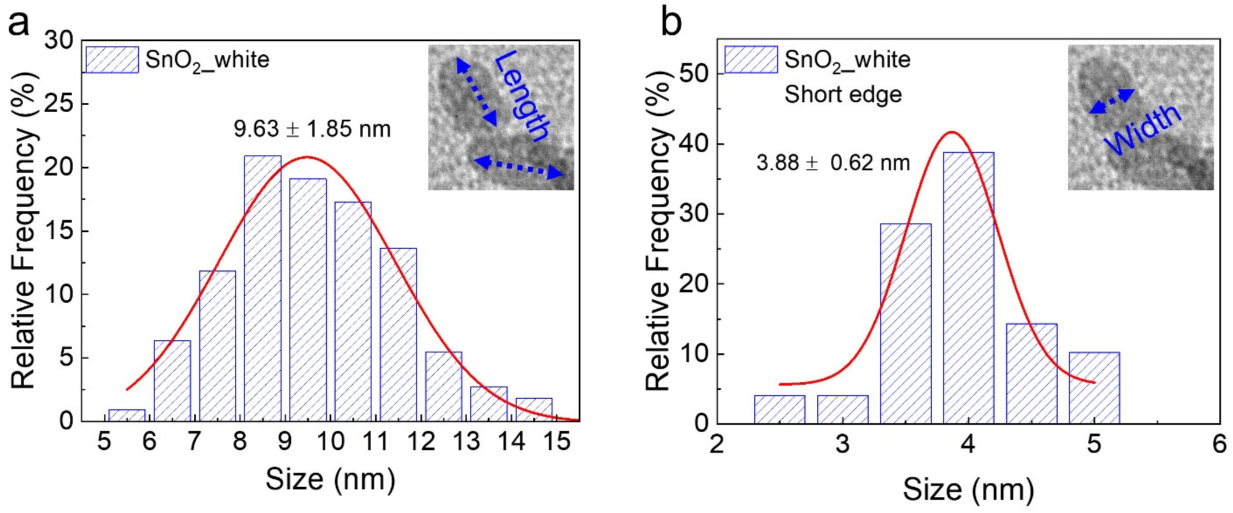

Figure S4. Size distribution histograms of the rod-like SnO_2 sample (SnO_2_white): (a), based on edge length, and (b), based on width.

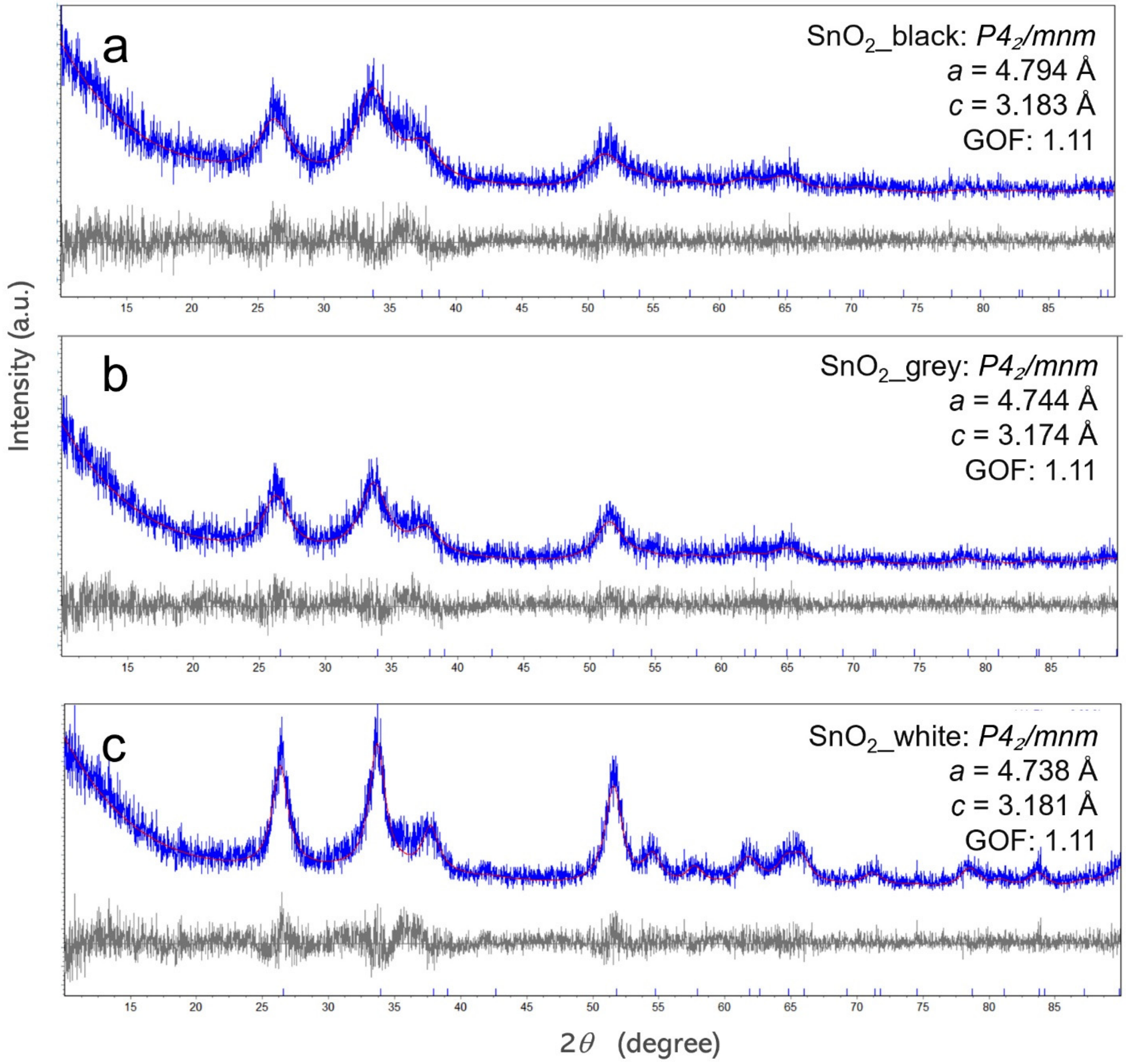

Figure S5. Lattice parameters of (a) SnO_2_black, (b) SnO_2_grey, and (c) SnO_2_white obtained from Pawley fitting. "GOF" refers to the goodness of fit. (The unit cell parameters of bulk SnO_2 cassiterite, JCPDS-ICDD card 41-1445, are a = 4.738 Å, c = 3.187 Å).

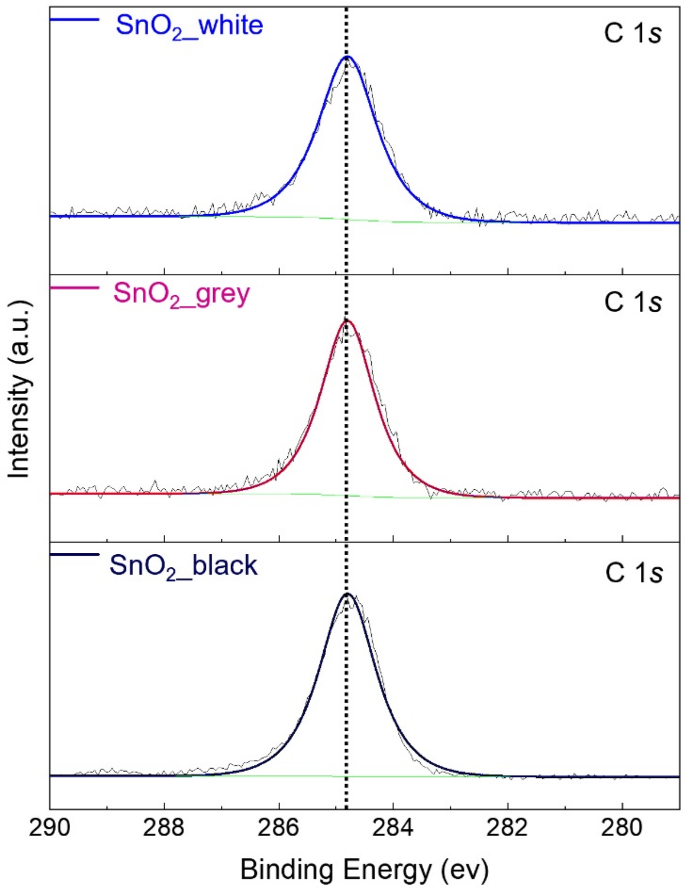

Figure S6. XPS spectra of C 1*s* for the three as-synthesized SnO_2 samples: SnO_2_white, SnO_2_grey, and SnO_2_black.

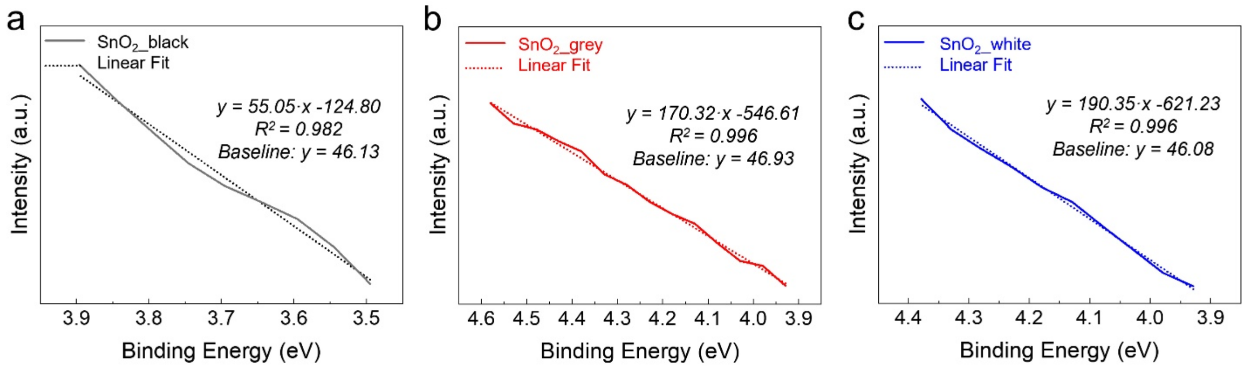

Figure S7. Extrapolation of the straight linear regions (3.9 eV to 3.5 eV) to the baseline for the three samples, (a) SnO_2_black, (b) SnO_2_grey, and (c) SnO_2_white, respectively, yielding the maximum energy of the valence band (E_V) for each.
